# Neural minimization methods (NMM) for solving variable order fractional delay differential equations (FDDEs) with simulated annealing (SA)

**DOI:** 10.1371/journal.pone.0223476

**Published:** 2019-10-10

**Authors:** Amber Shaikh, M. Asif Jamal, Fozia Hanif, M. Sadiq Ali Khan, Syed Inayatullah

**Affiliations:** 1 Department of Humanities and Sciences, National University of Computer and Emerging Sciences, Karachi, Pakistan; 2 Department of Basic Sciences Federal Urdu University of Art, Science and technology Karachi & Cadet College, Karachi, Pakistan; 3 Department of Mathematics, University of Karachi, Karachi, Pakistan; 4 Department of Computer Sciences, University of Karachi, Karachi, Pakistan; 5 Department of Mathematics, University of Karachi, Karachi, Pakistan; Universite de Bordeaux, FRANCE

## Abstract

To enrich any model and its dynamics introduction of delay is useful, that models a precise description of real-life phenomena. Differential equations in which current time derivatives count on the solution and its derivatives at a prior time are known as delay differential equations (DDEs). In this study, we are introducing new techniques for finding the numerical solution of fractional delay differential equations (FDDEs) based on the application of neural minimization (NM) by utilizing Chebyshev simulated annealing neural network (ChSANN) and Legendre simulated annealing neural network (LSANN). The main purpose of using Chebyshev and Legendre polynomials, along with simulated annealing (SA), is to reduce mean square error (MSE) that leads to more accurate numerical approximations. This study provides the application of ChSANN and LSANN for solving DDEs and FDDEs. Proposed schemes can be effortlessly executed by using Mathematica or MATLAB software to get explicit solutions. Computational outcomes are depicted, for various numerical experiments, numerically and graphically with error analysis to demonstrate the accuracy and efficiency of the methods.

## Introduction

In the old days, fractional calculus was only used by pure mathematicians due to its imperceptible applications at that time. When mathematicians were trying to implement fractional calculus in modeling of physical phenomena then applicability of this marvelous tool was successfully revealed. Therefore, in recent years, fractional derivatives have been used in many phenomena in electromagnetic theory, fluid mechanics, viscoelasticity, circuit theory, control theory, biology, atmospheric physics, etc. Many real-world problems can be accurately modeled by fractional differential equations (FDEs) such as damping laws, fluid mechanics, rheology, physics, mathematical biology, diffusion processes, electrochemistry, and so on.

Nowadays, there has been a tremendous increase of interest in theory of FDDEs, due to the fact that it can express the system of many dynamics population with more accuracy as demonstrated by past research in science and engineering. In the systems of real world, delays can be recognized and implemented everywhere and due to this advancement in modeling it has captured a lot of attention of the scientific community towards it.

### Literature review

Many researchers were inspired by the subject of fractional order and addressed these phenomena in different scenarios. For example, Lie et al. [1] used phase portraits, time domain waveform and bifurcation to examine the behavior of nonlinear system of fractional order. Modified Kalman filter was proposed by [2] to deal with fractional order system. To achieve the suitable generalized projective synchronization (GPS) of incommensurated fractional order system,[3] has introduced fuzzy approach. Coronel-Escamilla et al.[4] used Euler–Lagrange and Hamilton formalisms for describing the the fractional modeling and control of an industrial selective compliant assembly robot arm(SCARA).

Many researchers have made their efforts to investigate DEs and FDEs such as Zhang et al. [5] studied generalized Burgers equation and a generalized Kupershmidt equation through lie-group analysis method for similarity reductions and exact solutions, Zhang and Zhou [6] also carried out analysis of Drinfeld-Sokolov-Wilson system through symmetry analysis method, Yang et al.[7, 8] implemented travelling wave solution to local fractional two-dimensional Burgers-type equations and Boussinesq equation in fractal domain. Atangana and Gómez-Aguilar [9] presented exact solution and semi group principle for evolutions equation by using three different definitions of fractional derivatives. Lie et al. [10] calculated the Haar wavelet operational matrix together with Block phase function to find the solution of FDEs. Li et al. [11] have also made a successful attempt to approximate the same problem by using Chebyshev wavelet operational matrix method. Adomian decomposition method and variational iteration method were implemented in [12–16] to solve a variety of FDEs.While differential transform method and power series method are also noteworthy for the solution of FDEs [17–22].

In recent years the approach of neural architecture has been used to solve Des and FDEs. In [23] Aarts and Veer implemented the multilayer neural algorithm for the solution of partial differential equations (PDEs) with evolutionary algorithm for training of weights. Feed forward NN technique, with the blend of piece splines of Lagrange polynomial, was proposed by [24]. Same approach was applied by [25] in which genetic algorithm was used as an evolutionary algorithm for training of network of nonbed-catalytic gas reactor system. For solving PDEs [26] has put an effort by implementing NN together with Broden-Flecher-Goldfarb-Shanno algorithm. Nelder Meade optimization procedure with hybrid neural network (HNN) was adopted by [27] for the numerical simulation of higher order DEs. Levenberg-Marquardt algorithm with ANN and Mittag-Leffler kernel was implemented by [28] to solve FDEs.

Those systems that are governed by their past are modeled in the form of delay differential equations.DDEs are proved useful in control systems [29], lasers, traffic models [30], metal cutting, epidemiology, neuroscience, population dynamics [31], and chemical kinetics [32] condition. Due to the infinite dimensionality of delay systems it is very challenging to analytically analyze DDEs, therefore numerical simulations of DDEs play a key role for study of such systems. A noteworthy study of FDDEs through neural network can be visualized in [33] Existence and uniqueness theorems on FDDEs are discussed in [34–36]

This study will generate an approximate solution for solving the DDEs and FDDEs by using ChSANN and LSANN, which were first developed by Khan et al. [37,38] to solve Lane Emden equations and fractional differential equations on a discrete domain. In the following paper we have developed an approach to solve the higher order DDEs and FDDEs on a continuous domain. This paper is organized as follows: first section of paper describes introduction and literature review while second section concerns with details of the methodologies with well explained algorithm and implication procedure. Error analysis procedure is explained in third section where as fourth, fifth and sixth sections describe the numerical experiments along with results and their discussions.

### Methodology

The proposed methodologies are based on functional link neural network with optimization through thermal minimization. In this study, the Caputo definition will be used for working out the fractional derivative in the subsequent procedure. These definitions of commonly used fractional differential operators are discussed in [39].

Initially introduced by Pao [40], ChSANN and LSANN are the revised version of functional link artificial NN coupled with optimization strategy for learning. Functional link architecture of NN was design to build the connection between the linearity in a single layer NN and the computationally challenging multilayer NN.

Inspired by the physical process of annealing, SA is basically a kind of combinatorial optimization process. This process is based on two steps: first to perturb and then to measure quality of the solution. MSE is basically the fitness function denoted by *E*_*r*_, that can be minimized by the use of SA.

### Algorithm

Due to the structural similarity in ChSANN and LSANN, the steps of algorithm are described in combination for both methods. Accuracy of results depends on the selection of base polynomial.

**Step 1:** Initialize the network by applying number of Chebyshev polynomials or Legendre polynomials (to independent variable *x*) *k* = *0 to n*.

**Step 2**: Provide in each polynomial a network adaptive coefficient (NAC).

**Step 3**: Calculate the summation of product of NAC and Chebyshev polynomial or Legendre polynomial and store the value in *ϕ* or *ψ* respectively.

**Step 4:** Activate *ϕ* and *ψ*, by first three terms of Taylor Series expansion of *tanh* function.

**Step 5:** As given by Lagaris and Fotiadias [41] trial solution will be generated by the help of initial conditions and activated *ϕ* (in case of LSANN *ψ*)

**Step 6:** Calculate the value of delay trail solution by repeating step 1 to 5 with delay independent variable.

**Step 7:** Calculate the MSE of DDEs or FDDEs by discretizing the domain in *β* number of points.

**Step 8:** Set Tolerance for accepting minimized value of MSE.

**Step 9:** Minimize the MSE by thermal minimizing methodology with the following **settings** from Mathematica 11.0.

➢ Level iterations→50
➢ Perturbation Scale→1.0
➢ Probability function→ $e^{\frac{-Log(i+1)\nabla MSE}{T}}$
➢ Random seed→ 0
➢ Tolerance for accepting constraint violations→ 0.001

**Step 10**: If the value of MSE falls in the range of pre-defined criteria, then substitute the value of NAC in trial solution to get the output otherwise go to step 1 change the value of *n* and repeat whole procedure till the acceptable MSE is obtained.

**Fig 1:**
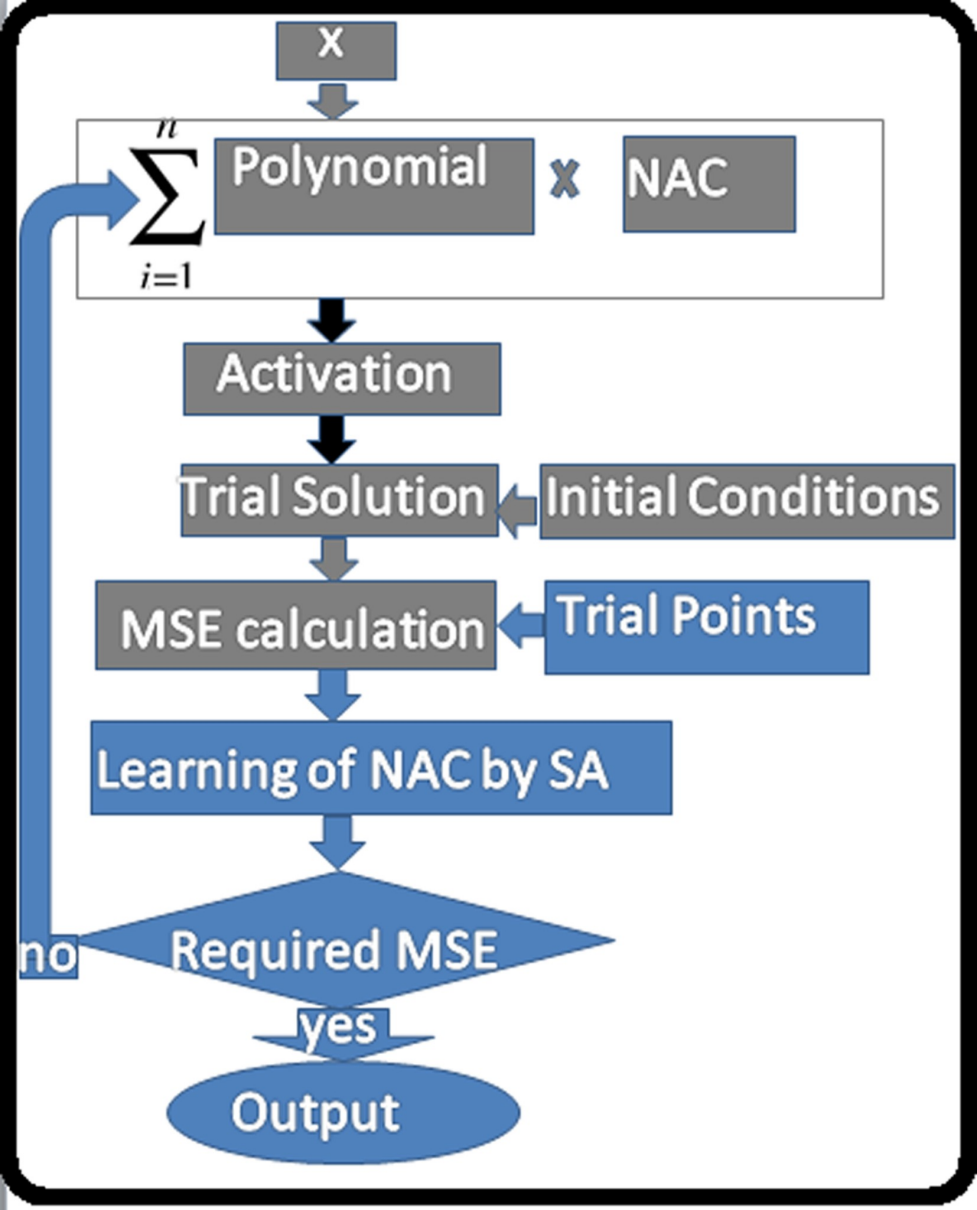
Pictorial presentation of algorithm.

Pictorial presentation of above algorithm can be observed in Fig 1

### Employment on delay differential equation

Now we apply ChSANN and LSANN on DDEs of the following type,

$$f^{\left(n\right)}\left(x\right)=F\left(f\left(x-\tau x\right),f^{\prime }\left(x\right),f^{\prime \prime }\left(x\right),f^{\prime \prime \prime }\left(x\right),\ldots ,f^{\left(n-1\right)}\left(x\right)\right)+g\left(x\right).$$ (1)

With initial conditions as follows,

$$f\left(0\right)=\alpha_{0,}\;f^{\prime }\left(0\right)=\alpha_{1,}\;\ldots f^{\left(n-1\right)}\left(0\right)=\alpha_{n-1}.$$ (2)

For implementation of ChSANN and LSANN Eq (1) can be written as

$$f^{\left(n\right)}\left(x\right)-F\left(f\left(x-\tau x\right),f^{\prime }\left(x\right),f^{\prime \prime }\left(x\right),f^{\prime \prime \prime }\left(x\right),\ldots ,f^{\left(n-1\right)}\left(x\right)\right)-g\left(x\right)=0.$$ (3)

For trial and delay trial solution of above differential equation consider,

$$\phi =\sum_{k=1}^{m}w_{k}T_{k-1}\left(x\right),$$ (4)

where *T*_*k*_ is Chebyshev polynomial with the following recursive formula defined as

$$T_{k+1}\left(x\right)=2xT_{k}\left(x\right)-T_{k-1},\;k\geq 2$$

Here *T*_*0*_ = *1* and *T*_*1*_ = *x* are the fundamental values of Chebyshev polynomials and

$$\psi =\sum_{j=1}^{n}w_{k}\;L_{j-1}\left(x\right),$$ (5)

where *L*_*j*_ is Legendre polynomial with the following recursive formula defined as:

$$L_{j+1}\left(x\right)=\frac{1}{\left(j+1\right)}(2j+1)\;x\;L_{j}(x)-\frac{1}{\left(j+1\right)}j\;L_{j-1}(x),\;j\geq 2,$$ (6)

where, *L*_*0*_(*x*) = *1* and *L*_*1*_(*x*) = *x* are the fundamental values of Legendre polynomials.

Here we are using first three terms of Taylor series expansion of *tanh* function to activate *ϕ* and *ψ*. As defined by Lagaris and Fotiadis [41], the trial solution of Eq (1) can be written as,

$$f_{trial}\left(x,w\right)=\alpha_{0}+\alpha_{1}x+\frac{x^{2}}{2!}\alpha_{2}+\ldots +N\left(x,w\right)\frac{x^{k}}{k!}.$$ (7)

Where *N* is the activated *ϕ* or *ψ*, depends on the method, while the delay trial solution can be given by replacing the *x* by *x*−*τx*.

The MSE of the Eq (1) will be calculated from the following:

$$E_{r}\left(w\right)=\sum_{l=1}^{\beta }{\left(f_{trial}{}^{\left(n\right)}\left(x_{l},w\right)-F\left(\begin{array}{l} f_{trial}\left(x_{l},w\right),f_{trial}\left(x_{l}-\tau x_{l},w\right),f_{trial}^{\prime }\left(x_{l},w\right),f_{trial}^{\prime \prime }\left(x_{l},w\right)\\ ,\ldots ,f^{\left(n-1\right)}{}_{trial}\left(x_{l},w\right) \end{array}\right)-g\left(x_{l}\right)\right)}^{2}$$ (8)

here *β* represents the number of trial points while Eq (8) will be the fitness function for learning of NAC. For implementation on FDDEs only the method of computing the fractional derivative of trial solution will vary, which will be taken in this study according to Caputo definition as follows.

### Definition

According to [39] Caputo operator for *λ*>0 can be defined as:

$$D^{\lambda }g\left(\eta \right)=\frac{1}{\Gamma \left(m-\lambda \right)}\int_{0}^{\eta }{\left(\eta -t\right)}^{m-\eta -1}g^{\left(n\right)}\left(t\right)dt,\;m-1<\lambda <m,m\in N,$$

with

- *D*^*λ*^*c* = 0, where *c* is a constant
- $D^{\lambda }\left(\eta^{\beta }\right)=\{\begin{array}{l} 0,\;\lambda \in N_{0},\beta <\lambda \\ \frac{\Gamma \left(\beta +1\right)}{\Gamma \left(\beta +1-\lambda \right)}\eta^{\beta -\lambda },\;\mathrm{otherwise} \end{array}$

Mathematica 11.0 is the minimization implementation tool in this study but details can be seen from [42].

### Error analysis

The error analysis of numerical experiments for ChSANN and LSANN methodologies can be observed by following procedure. By substituting the values of NAC, after learning from SA algorithm, into the trial solution it will become ChSANN or LSANN solution that can be further substituted into Eq (9) for analyzing the accuracy of method on the domain of [0,1].

$$E\left(x\right)=\left|f^{n}\left(x\right)-F\left(f\left(x-\tau x\right),f^{\prime }\left(x\right),f^{\prime \prime }\left(x\right),f^{\prime \prime \prime }\left(x\right),\ldots ,f^{\left(n-1\right)}\left(x\right)\right)-g\left(x\right)\right|≅0$$ (9)

While *f*(*x*) is the obtained approximated continuous solution by ChSANN or LSANN. *E*_*r*_(*x*_*i*_) tends to 0 as the value of MSE obtained by ChSANN and LSANN is in the predefined range. Convergence of solution is totally dependent on the learning methodology of respected NN architecture which is SA in the current case.

### Numerical experiments

#### Experiment 1

Consider 2^nd^ order DDE along with the initial conditions as:

$$y^{\left(\alpha \right)}\left(x\right)=\frac{3}{4}y\left(x\right)+y\left(\frac{x}{2}\right)-x^{2}+2,\;y\left(0\right)=0;y^{\prime }\left(0\right)=0;\alpha =2.$$

The exact solution when

*α* = *1* is given as:

$$y\left(x\right)=x^{2}$$

**Fig 2:**
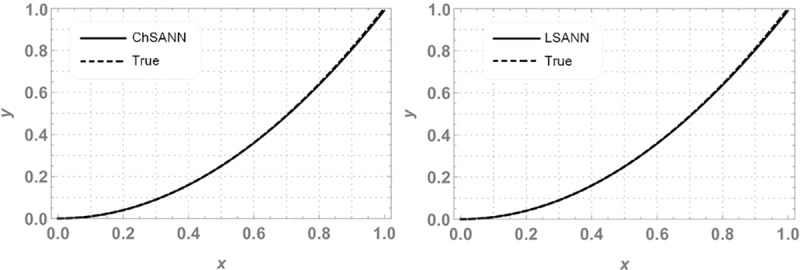
Comparison of ChSANN and LSANN with true solution.

**Fig 3:**
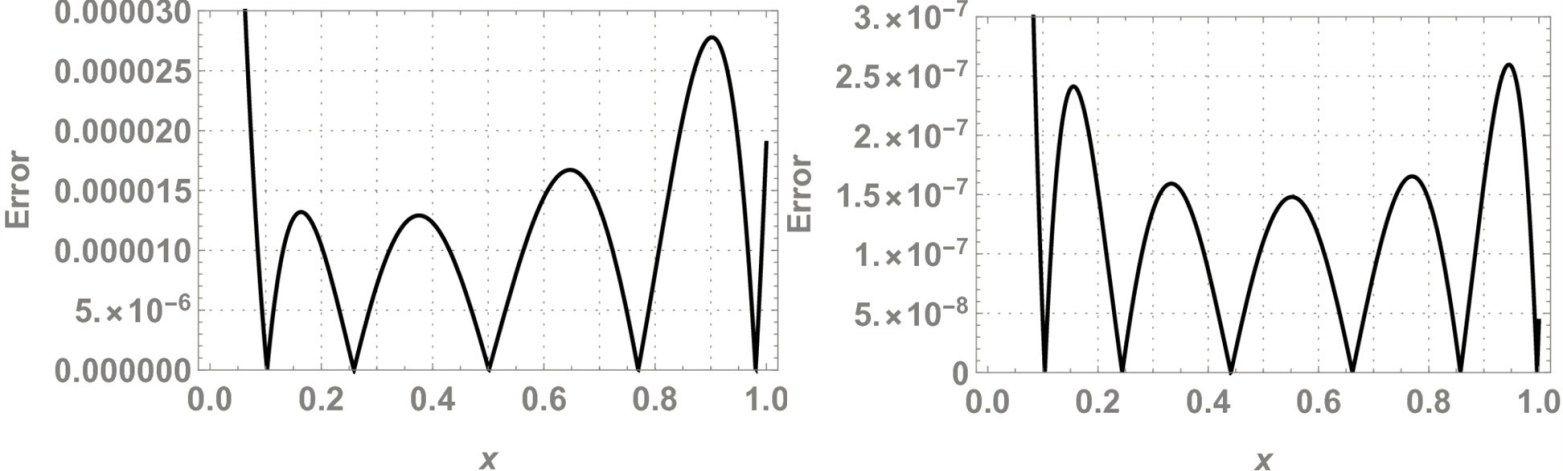
Error analysis of ChSANN and LSANN.

In this experiment we employed proposed methodologies on above second order linear DDE on domain of [*0*,*1*].Both the methods were employed by dividing the domain with 10 equidistant training points and 6 NAC. For ChSANN and LSANN at *α* = 2, the MSE at defined conditions is found to be *1*.*89855* ×*10*^*−11*^ and *1*.*32344* ×*10*^*−14*^ respectively. (Fig 2) depicts the comparison of both methods with true solution at continuous domain of [0,1], while (Fig 3) displays the error analysis for both methods at *α* = 2. For the above experiment the trial and delay trail solutions are found to be following:

$$y_{trial}=\frac{x^{2}}{2}N$$

and

$$y_{dtrial}=\frac{{\left(x/2\right)}^{2}}{2}M.$$

**Fig 4:**
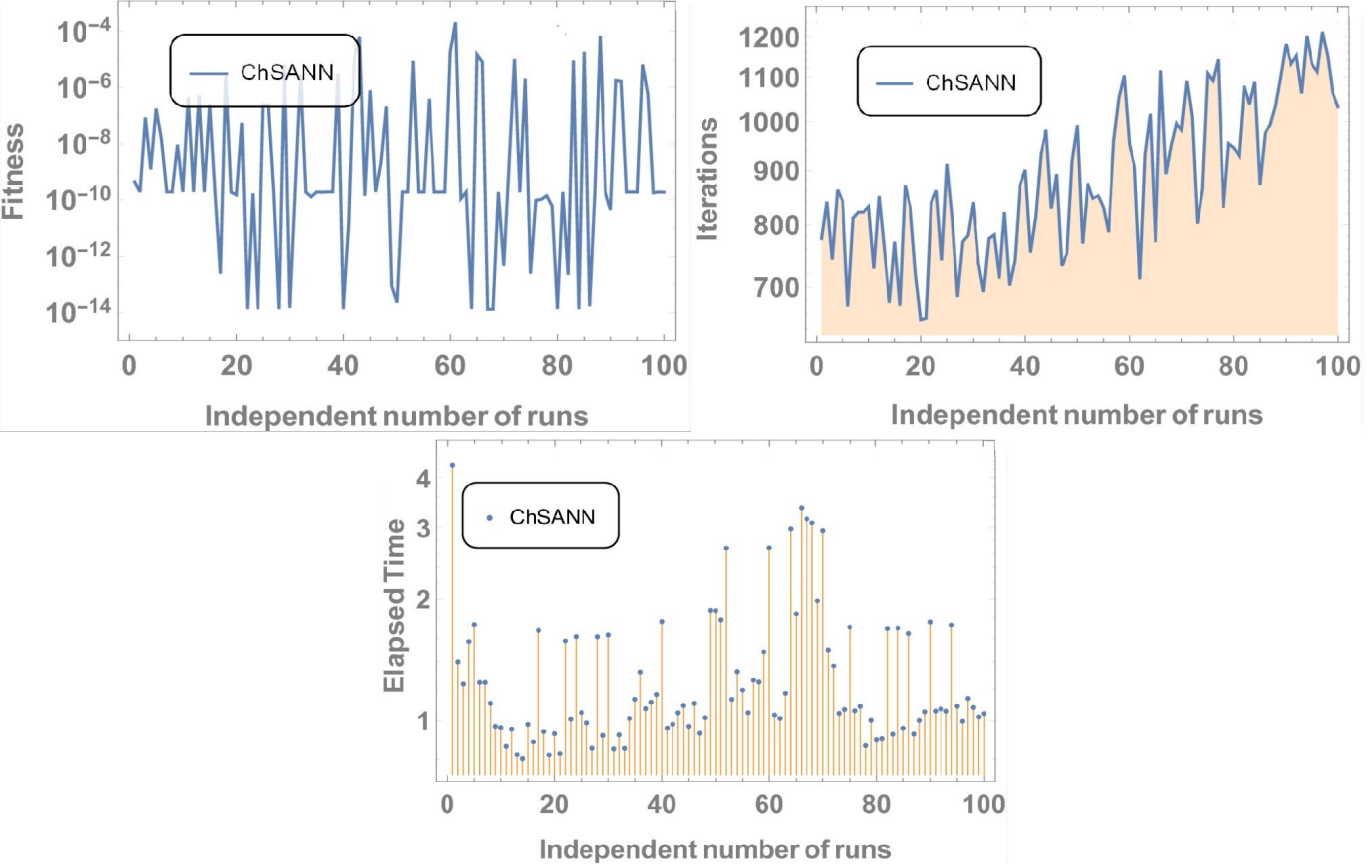
Results for 100 number of independent runs.

**Fig 5:**
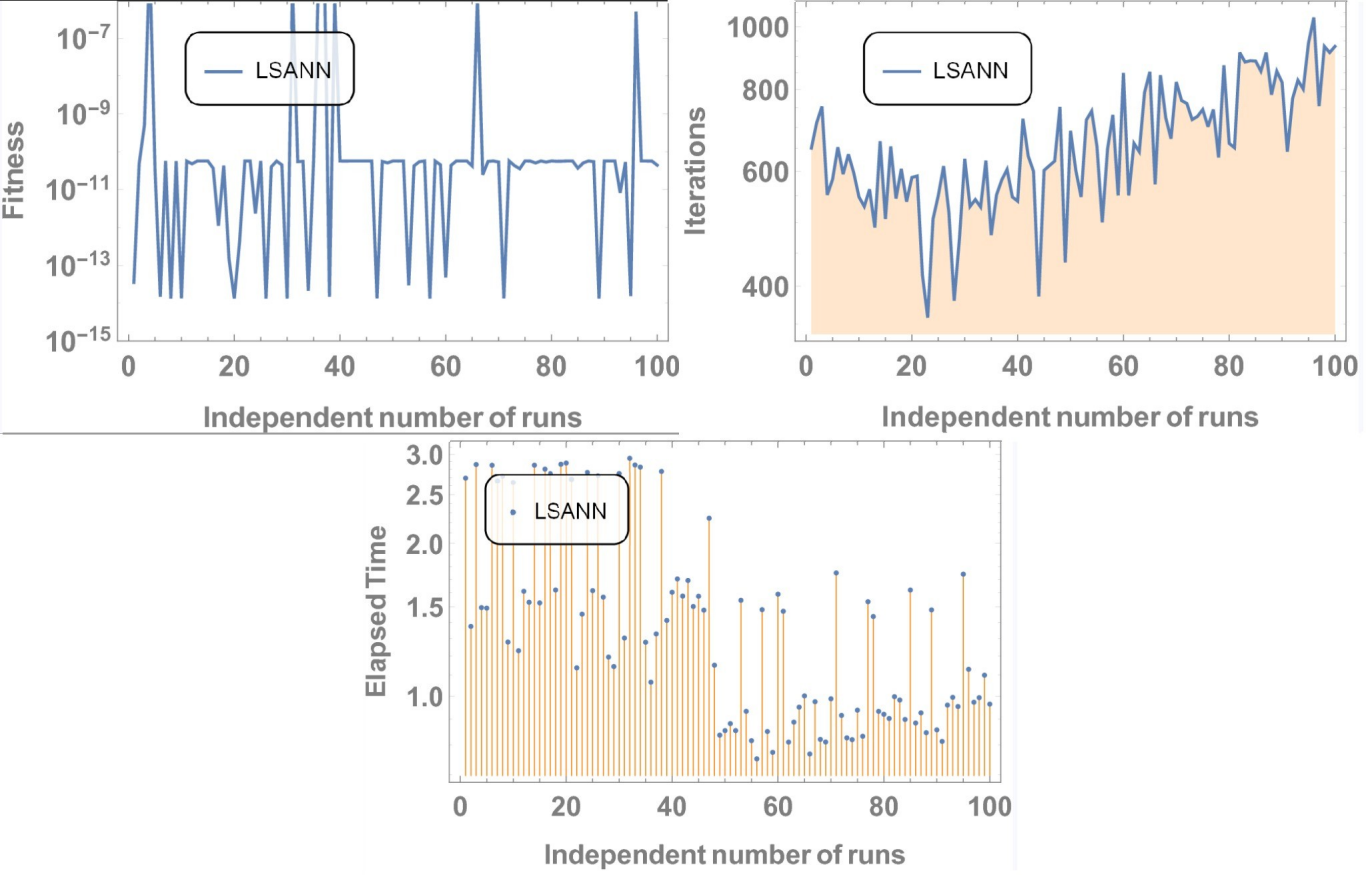
Results for 100 independent runs.

Where, *N* and *M* are the structural outputs of both NNs. Table 1 displays the final values of NAC after training by SA algorithm and Figs 4 and 5 is displaying the data for 100 independent runs by altering the scale for random jumps.

**Table 1:** ChSANN and LSANN results.

SNo	NAC	ChSANN	LSANN
1	*w*_1_	1.70693523	1.710066834
2	*w*_2_	-0.00101189	-0.00004054
3	*w*_3_	-0.00220052	-0.00372515
4	*w*_4_	-0.00028488	-0.00002618
5	*w*_5_	0.000058430	-0.00003209
6	*w*_6_	-0.00001175	-0.00002803

#### Experiment 2

Consider 3^rd^ order nonlinear DDE along with the initial condition as:

$$y^{\left(\alpha \right)}\left(x\right)=2y^{2}\left(\frac{x}{2}\right)-1,\;y\left(0\right)=0,\;y^{\prime }\left(0\right)=1,y^{\prime \prime }\left(0\right)=0,\;\alpha =3$$

The exact solution when *α* = *3* is given below:

y(x)=Sin(x)

**Fig 6:**
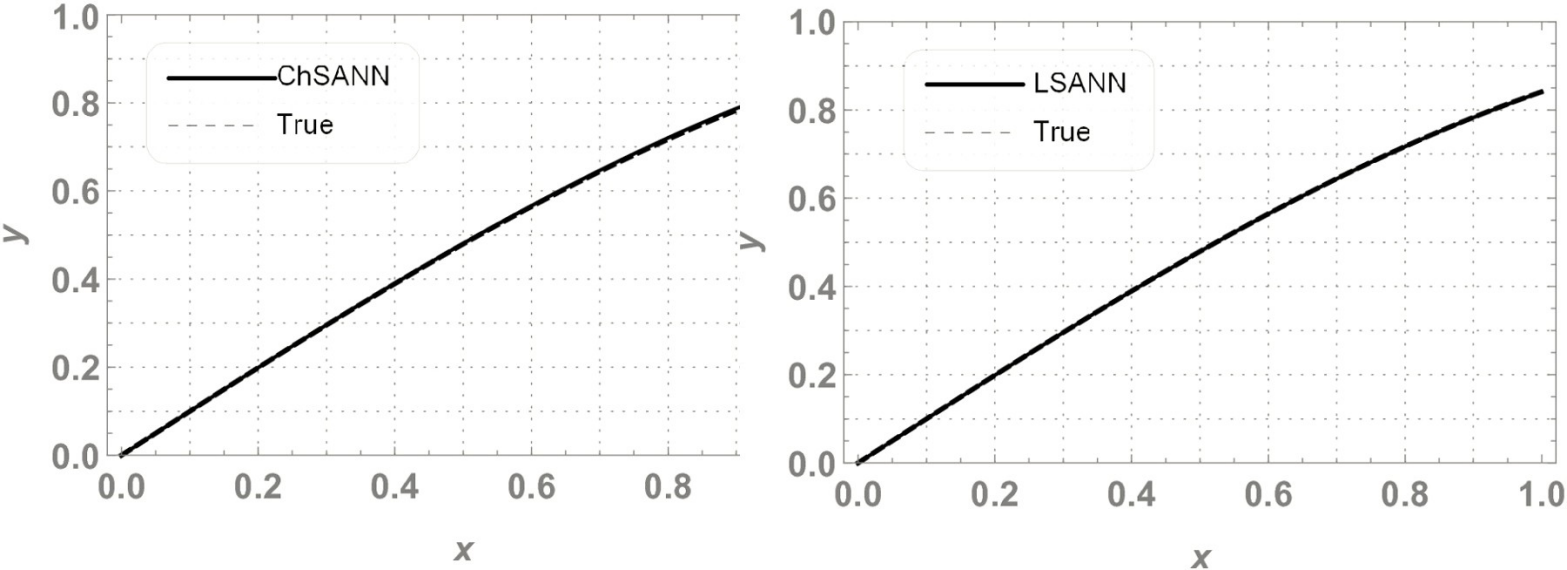
Comparison of ChSANN and LSANN with true solution.

**Fig 7:**
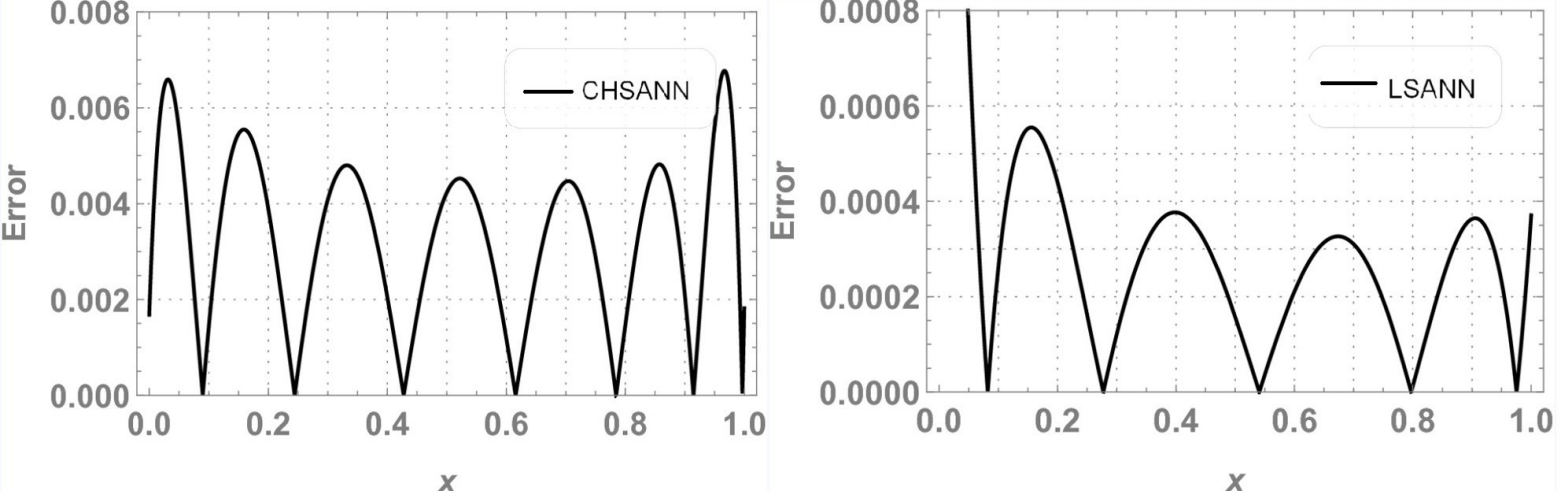
Error analysis of ChSANN and LSANN.

**Fig 8:**
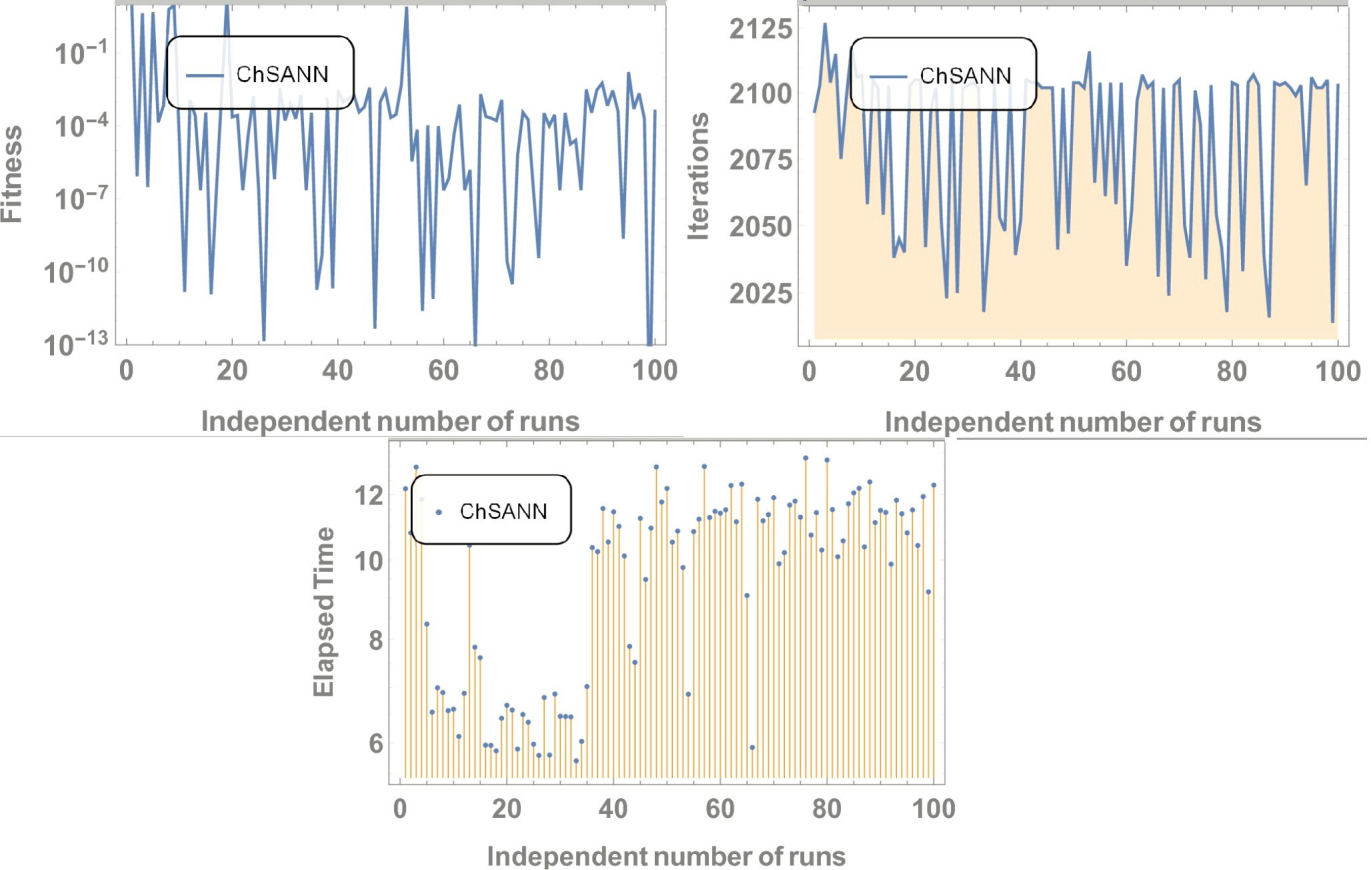
Results for 100 independent runs.

**Fig 9:**
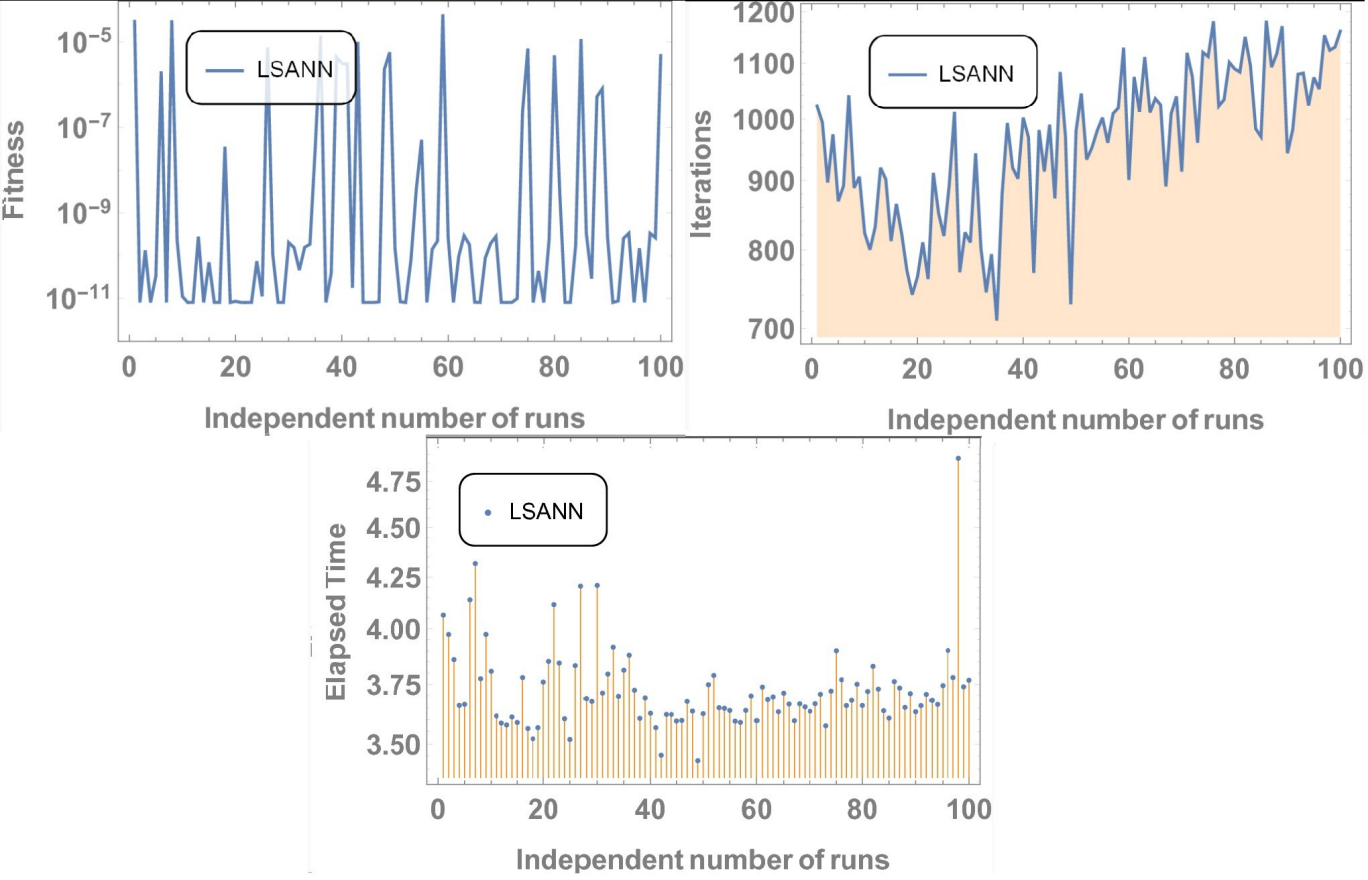
Results for 100 independent runs.

3^rd^ order nonlinear DDE is solved by ChSANN and LSANN on the continuous domain of [0,1]. ChSANN and LSANN were run for *10* and *6* NAC respectively while training points were *20* for both. With given predefined conditions MSE is found to be 2.5679×*10*^−*5*^ for ChSANN and *5*.*4843* ×*10*^−*7*^ for LSANN. Comparison of the methods with true solution can be visualized in Fig 6 and error analysis can be observed in Fig 7. Table 2 represents the final values of weights after training by SA algorithm while Figs 8 and 9 are displaying the results for 100 independent runs for elapsed time in seconds, fitness and number of iterations.

Following are trial and delay trail solution for the current experiment:

$$y_{trial}=x+\frac{x^{3}}{6}M$$

and

$$y_{dtrial}=\frac{x}{2}+\frac{{\left(x/2\right)}^{3}}{6}N.$$

While *M* and *N* are structural NN outputs of both the methods for in progress experiment.

**Table 2:** NAC values.

SNo	NAC	ChSANN	LSANN
1	*w*_1_	-0.874541110	-1.2104577306
2	*w*_2_	-0.478671752	-0.0384815109
3	*w*_3_	0.0165454550	0.06652279388
4	*w*_4_	0.4425599703	-0.0172612429
5	*w*_5_	- 0.594007418	0.00543949906
6	*w*_6_	0.4767150261	-0.00064910894
7	*w*_7_	-0.261651890	0.9618236111
8	*w*_8_	0.0976657350	-1.0110550149
9	*w*_9_	-0.022615861	2.3259303584
10	*w*_10_	0.0024649552	-2.0381716440

#### Example 3

Consider FDDE along with the initial conditions as:

$$y^{\left(\alpha \right)}\left(x\right)=\frac{1}{2}e^{\frac{x}{2}}y\left(x\right)+\frac{1}{2}y\left(x\right),\;y\left(0\right)=1,\;0\leq x\leq 1,\;0<\alpha \leq 1.$$

The exact solution at *α* = *1* is given by:

$$y\left(x\right)=e^{x}$$

**Fig 10:**
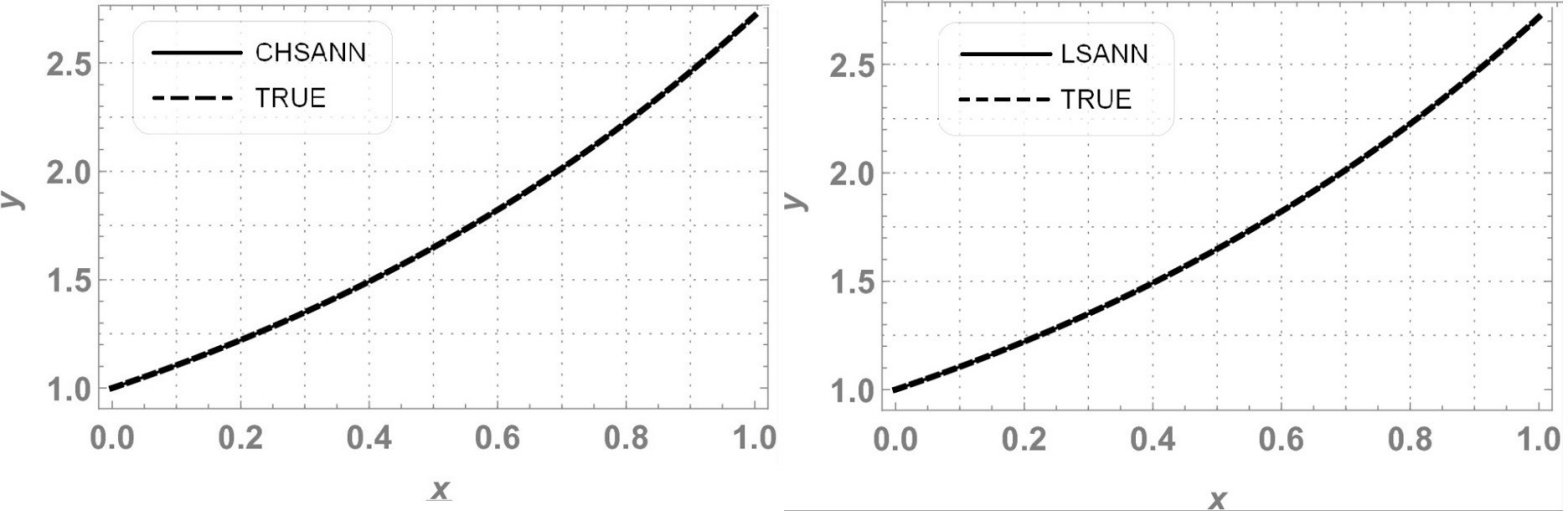
Comparison of ChSANN and LSANN with true solution at *α* = *1*.

**Fig 11:**
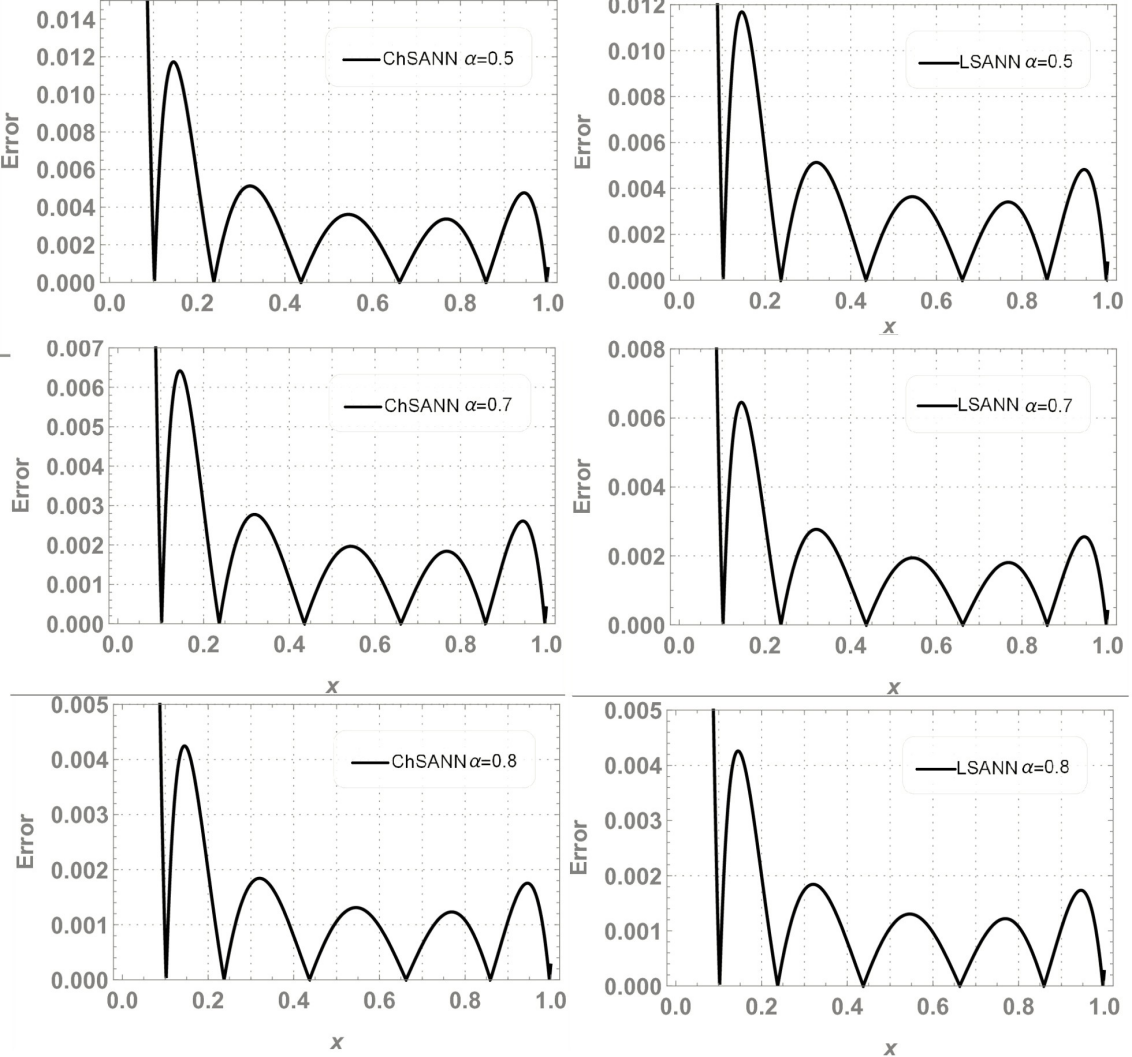
Error analysis.

**Fig 12:**
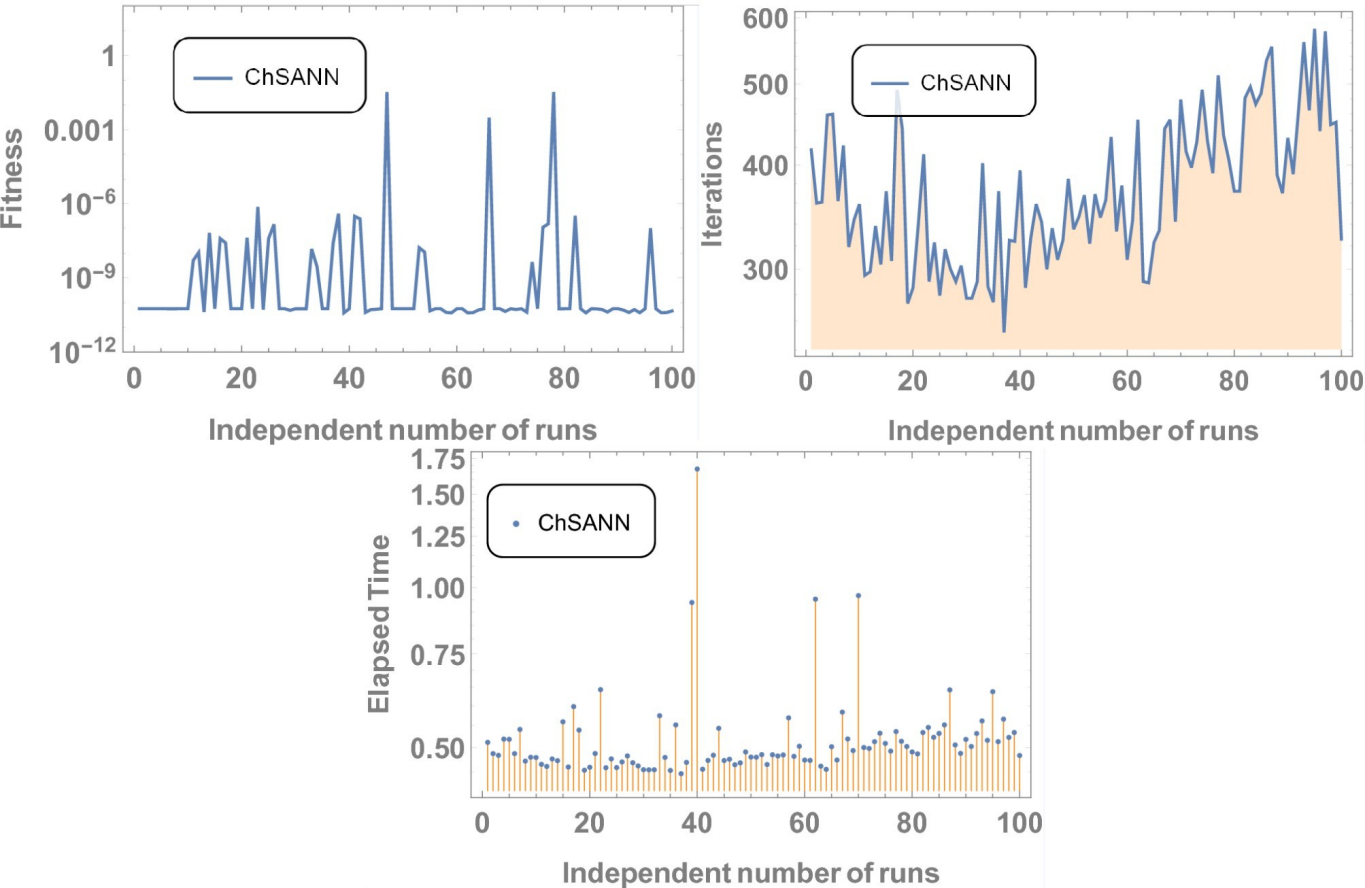
Results for 100 independent runs.

**Fig 13:**
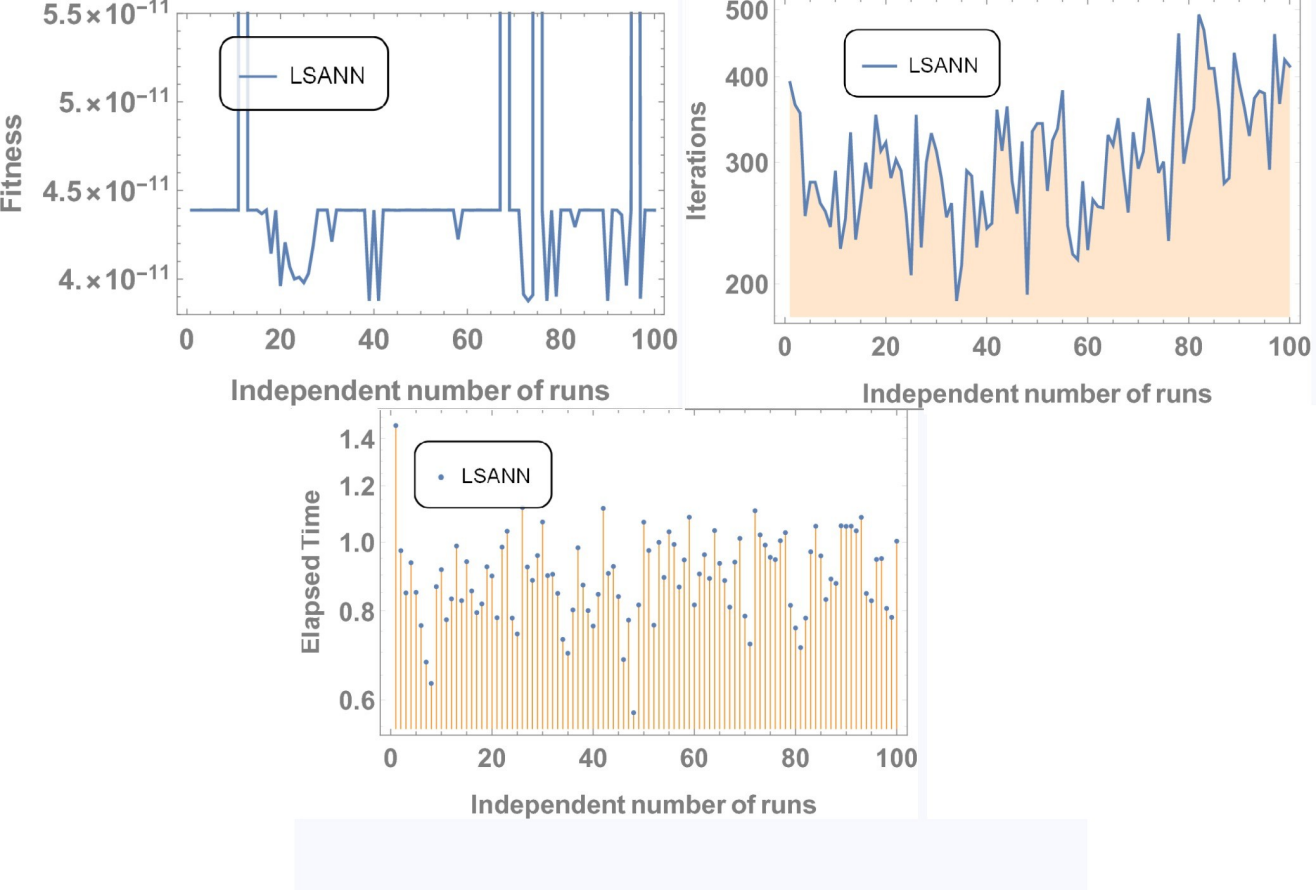
Results for 100 independent runs.

ChSANN and LSANN have been employed successfully on above FDDE with 10 training points and 6 NAC. (Fig 10) depicts the comparison of ChSANN and LSANN solutions with true values at *α* = *1* while values of final NAC after learning by SA algorithm can be visualized in Table 3. Both the methods were executed for different fractional values of *α* for which results can be visualized in Tables 4–6. Error analysis for all fractional values can be seen in (Fig 11) and Figs 12 and 13 is displaying the results for 100 independent runs for both the proposed methods

Trial and delay trial solutions are taken to be:

$$\begin{array}{l} y_{trial}=1+xN \end{array}$$

and

$$y_{dtrial}=1+\frac{x}{2}M.$$

**Table 3:** Values of NAC at *α* = *1*.

SNo	NAC	ChSANN	LSANN
1	*w*_1_	1.1669374428	1.1917375672
2	*w*_2_	0.5269817424	0.5257487109
3	*w*_3_	-0.079021346	-0.107447861
4	*w*_4_	0.0160510892	0.0265564411
5	*w*_5_	-0.0011808281	-0.003520220
5	*w*_6_	0.0000888300	0.000201124

**Table 4:** ChSANN and LSANN results at *α* = *0*.*5*.

X Values	ChSANN	LSANN
0.1	1.403383	1.403323
0.2	1.70248	1.702377
0.3	1.981465	1.981438
0.4	2.269594	2.269551
0.5	2.576318	2.576261
0.6	2.904964	2.904908
0.7	3.258834	3.258791
0.8	3.643645	3.643603
0.9	4.066385	4.066317
1.0	4.529678	4.529594
MSE For ChSANN	9.94644×10^-6^	
MSE For LSANN	9.947919×10^-6^	

**Table 5:** ChSANN and LSANN results at *α* = *0*.*7*.

X Values	ChSANN	LSANN
0.1	1.22611657	1.22608151
0.2	1.42562311	1.42559953
0.3	1.62462243	1.62460268
0.4	1.83454966	1.83452219
0.5	2.06067256	2.06063551
0.6	2.30584961	2.30580978
0.7	2.57260285	2.57256722
0.8	2.86419774	2.86416399
0.9	3.18461364	3.18456933
1.0	3.5369036	3.53690470
MSE For ChNN	2.86781×10^-6^	
MSE For LNN	2.86881×10^-6^	

**Table 6:** ChSANN and LSANN at *α* = *0*.*8*.

X Values	ChSANN	LSANN
0.1	1.17320141	1.1731957
0.2	1.33893581	1.3389313
0.3	1.50993727	1.5099335
0.4	1.69280737	1.6928027
0.5	1.89124814	1.8912421
0.6	2.10770277	2.1076959
0.7	2.34439824	2.3443915
0.8	2.60397962	2.6039730
0.9	2.88960028	2.8895924
1.0	3.20417343	3.2041639
MSE For ChSANN	1.2802637×10^−6^	
MSE For LSANN	1.28041×10^-6^	

#### Example 4

Consider Non-Linear FDDE along with the initial condition as:

$$y^{\left(\alpha \right)}\left(x\right)=1-2y^{2}\left(\frac{x}{2}\right),\;y\left(0\right)=1,\;y^{\prime }\left(0\right)=0,\;0\leq x\leq 1,\;1<\alpha \leq 2$$

The exact solution at *α* = 2 is given by:

y(x)=Cos(x)

**Fig 14:**
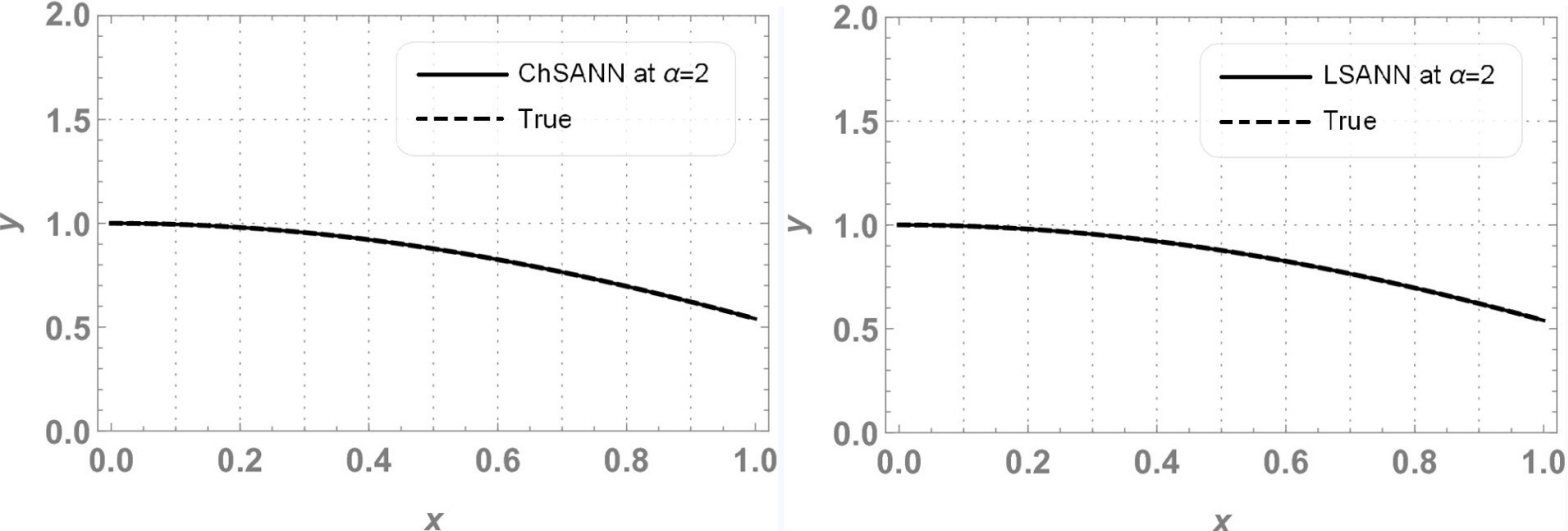
Comparison of ChSANN and LSANN with true values.

**Fig 15:**
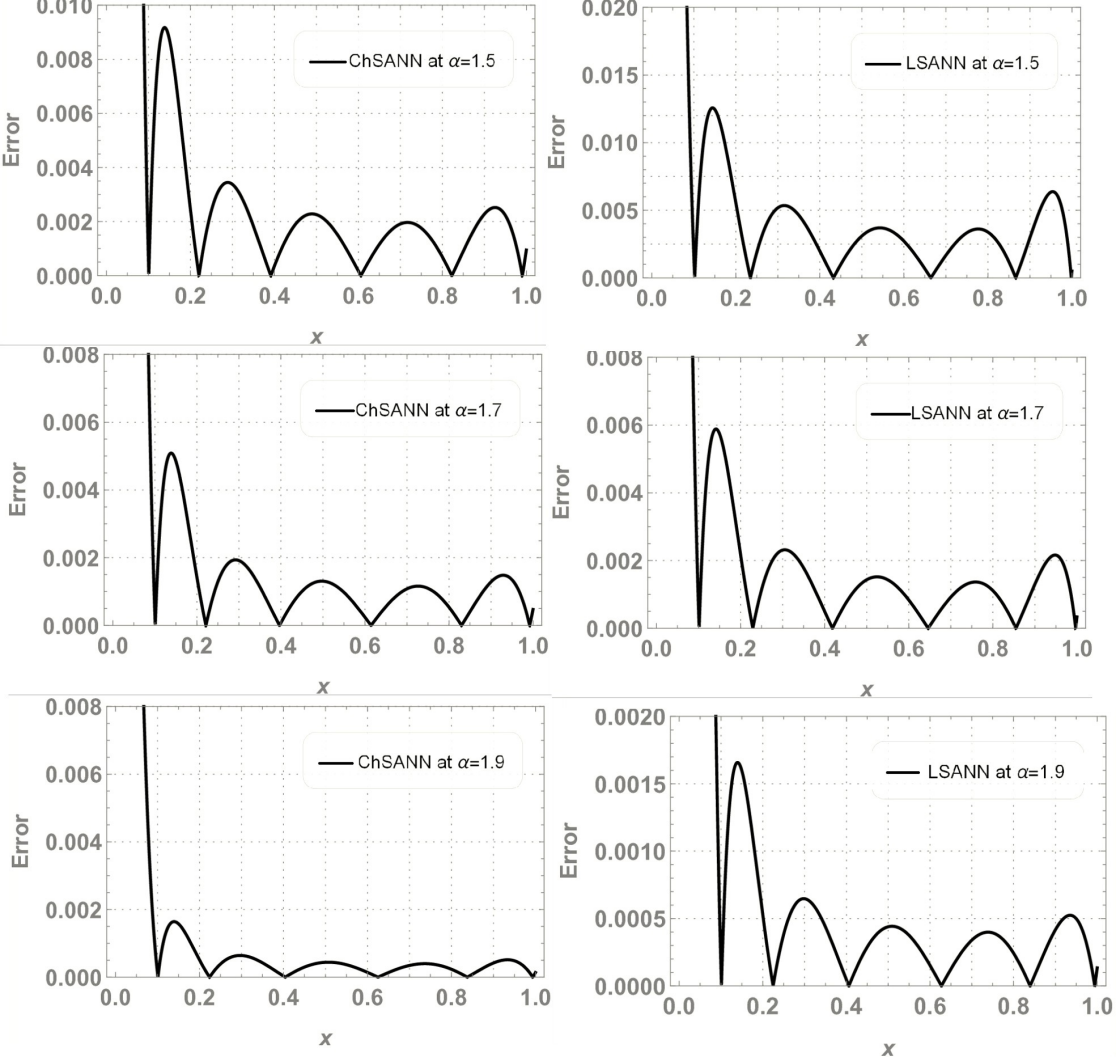
Error analysis.

**Fig 16:**
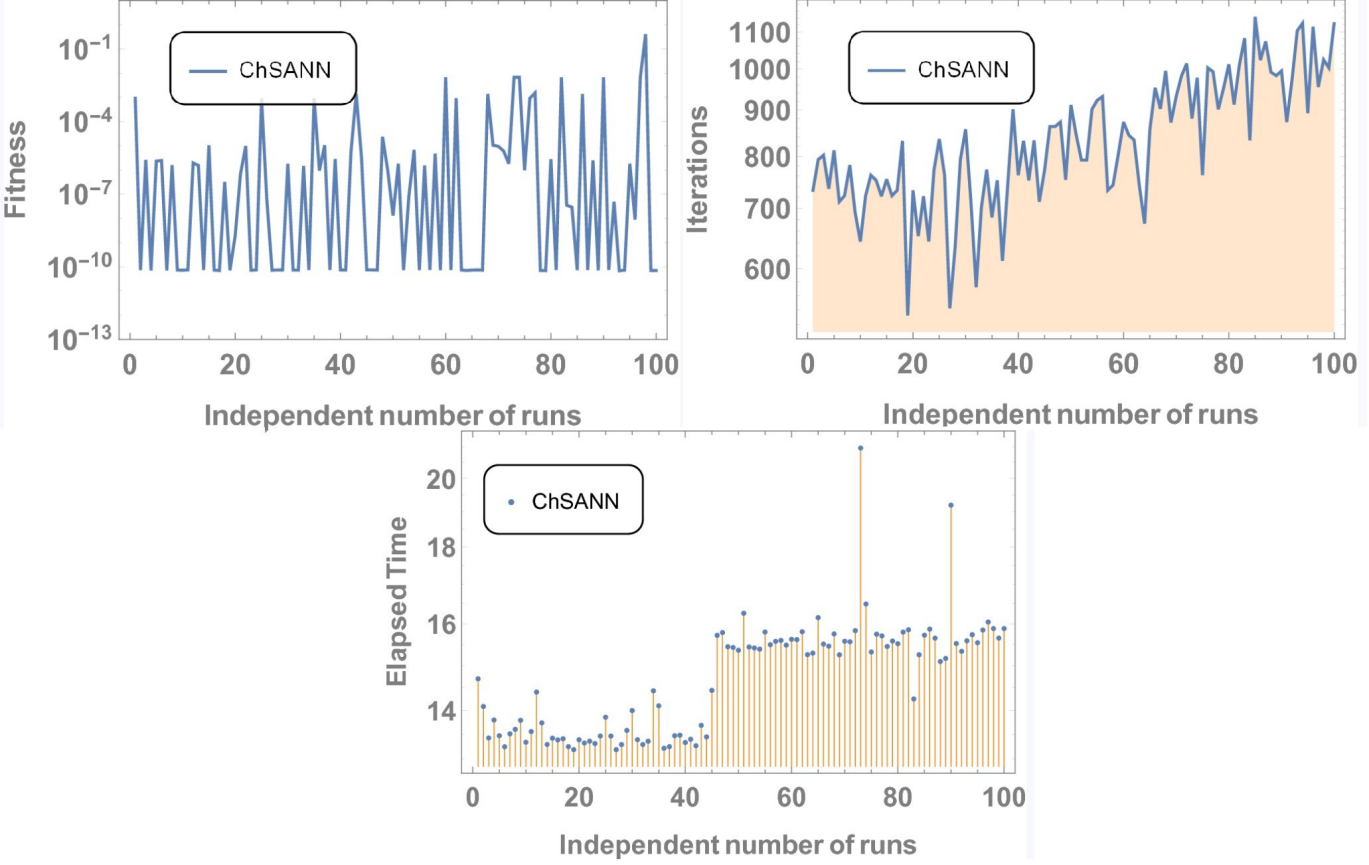
Results for 100 independent runs.

**Fig 17:**
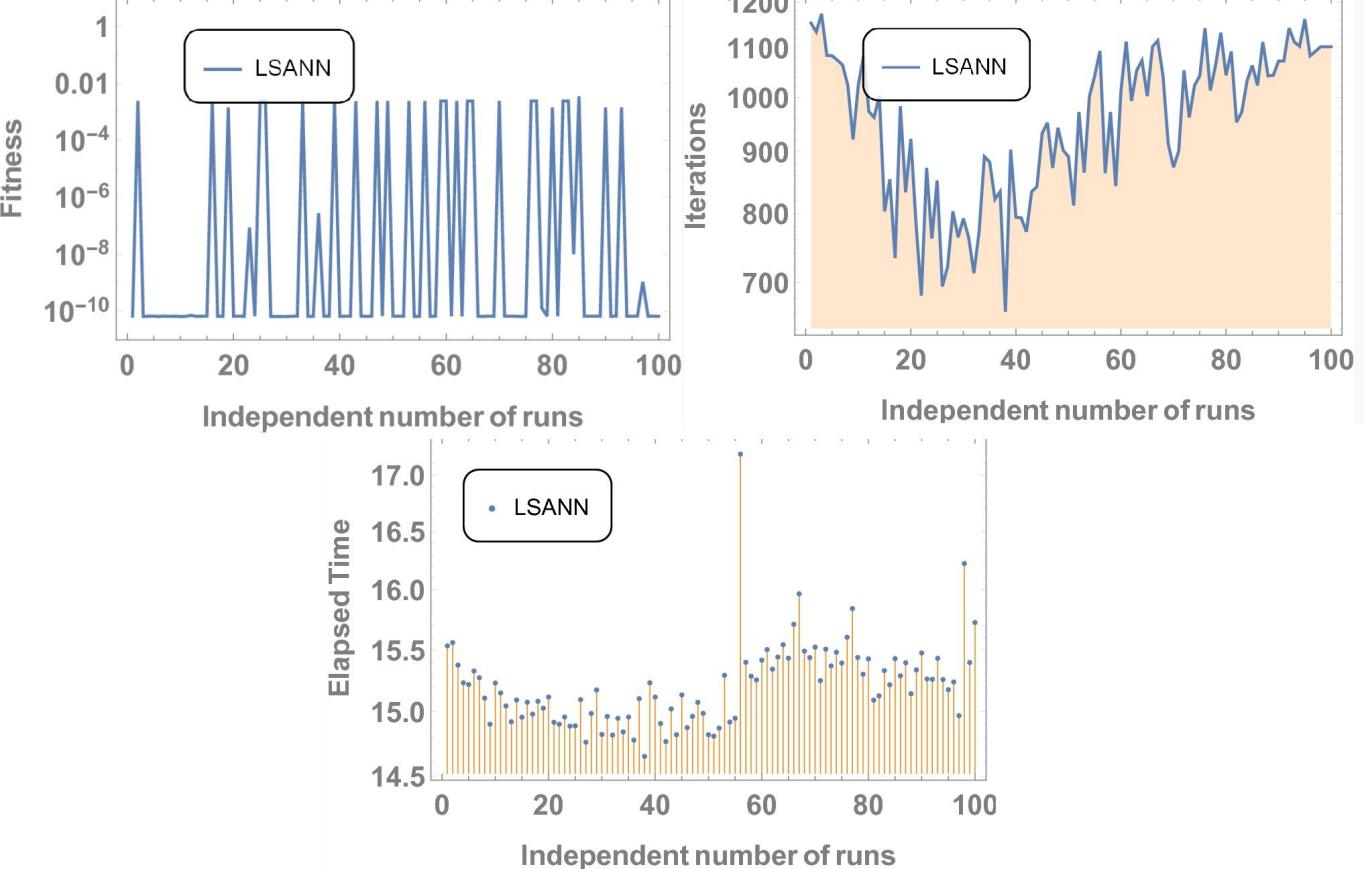
Results for 100 independent runs.

ChSANN and LSANN are implemented on the above experiment of nonlinear FDDE. (Fig 14) shows the comparison of both methods with true values at *α* = 2. While (Fig 15) represents the error analysis for different fractional values of *α*. Trial solution was taken in the same manner as in previous experiments. ChSANN and LSANN solutions with obtained MSE at different fractional values of *α* can be visualized in Tables 7–9 and Figs 16 and 17 are displaying the results for 100 independent runs.

**Table 7:** ChSANN and LSANN results for FDDE at *α* = *1*.*5*.

*x*	ChSANN	LSANN
0.1	0.9819344	0.9822488
0.2	0.9422867	0.9427229
0.3	0.8913654	0.8918057
0.4	0.8333956	0.8338889
0.5	0.7710079	0.7715448
0.6	0.7063767	0.7068606
0.7	0.6413646	0.6417259
0.8	0.5774801	0.5777550
0.9	0.5159194	0.5161458
1.0	0.4577416	0.4576439
MSE For ChSANN	3.32555×10^−6^	
MSE For LSANN	1.05097×10^−5^	

**Table 8:** ChSANN and LSANN results for FDDE at *α* = *1*.*7*.

*x*	ChSANN	LSANN
0.1	0.989296	0.9893501
0.2	0.962232	0.9623276
0.3	0.923181	0.9232965
0.4	0.874549	0.8746877
0.5	0.818103	0.8182648
0.6	0.755417	0.7555877
0.7	0.687973	0.6881368
0.8	0.617155	0.6173071
0.9	0.544234	0.5443786
1.0	0.470418	0.4705211
MSE For ChSANN	1.09296×10^−6^	
MSE For LSANN	1.73111×10^−6^	

**Table 9:** ChSANN and LSANN results for FDDE at *α* = *1*.*9*.

X Values	ChSANN	LSANN
0.1	0.9935924	0.99359236
0.2	0.9753719	0.97537194
0.3	0.9464374	0.94643741
0.4	0.9076268	0.90762677
0.5	0.8597139	0.87971386
0.6	0.8034944	0.80349434
0.7	0.7398116	0.73981153
0.8	0.6695533	0.66955325
0.9	0.5936416	0.59364162
1.0	0.5130313	0.51303120
MSE For ChSANN	1.26583×10^−7^	
MSE For LNN	1.2960433×10^−7^	

## Discussion

The above work is concerned with the successful implementation of ChSANN and LSANN on higher order DDEs and FDDEs. Some benchmark examples were considered for experimental cases and validity of implementation has been judged by standard error analysis procedure, data analysis of 100 number of runs of algorithm and comparison with other methods.

### Error analysis

For test experiment 1, MSE for ChSANN and LSANN for *α* = 2 were found to be *1*.*89855* ×*10*^*−11*^ and *1*.*32344* ×*10*^*−14*^ that gave the minimum error for ChSANN is *2*.*8* ×*10*^*−5*^ and for LSANN is *2*.*6* ×*10*^*−7*^ that can be easily visualized from (Fig 3). It shows that accuracy of both the methods is inversely proportional to the value of MSE and it can also be noticed that change of polynomials in both methods has strongly influenced the learning of NAC by SA algorithm that can be witnessed from Table 1. For experiment no 1, at similar conditions of training points and NAC, LSANN gave more promising results.

Similar trends can be depicted from the results of test experiment 2, in which MSE for ChSANN and LSANN for *α* = *3* were found to be *2*.*5679* ×*10*^*−5*^ and *2*.*20049* ×*10*^*−7*^. Error Analysis from (Fig 7) exhibiting the better performance of LSANN with less MSE as described above. Moreover it can also be visualized that this structure of neural network can be easily implemented on higher order nonlinear differential equations with ease.

In experiment no 3, both the proposed architectures were employed on linear delay fractional differential equation. MSE for ChSANN and LSANN at *α* = *1* were found to be *4*.*63009* ×*10*^*−11*^ and *3*.*88356*×*10*^*−11*^ respectively that gave excellent results that can be seen in (Fig 10). Further both the methods were also executed for *α* = *0*.*7*,*0*.*8 and 0*.*9*. Obtained MSE for all the fractional values obtained by both the methods were in the same range so the accuracy of results for all the fractional values is approximately similar that can be visualized in (Fig 11).

Experiment no 4 is a case of nonlinear FDDE. For *α* = *2* values of MSE with 6 NAC were found to be *7*.*82099* ×*10*^*−6*^ and *1*.*050097* ×*10*^*−5*^. For fractional values of *α* ChSANN is showing better results than LSANN at *α* = *1*.*5* and *α* = *1*.*9*, with better values of MSE for ChSANN and for *α* = *1*.*7* both the methods are exhibiting the similar accuracy. Accuracy at fractional values can be visualized from (Fig 15).

#### Data analysis for 100 numbers of independent runs

For each test experiment, algorithms of proposed techniques were executed 100 times by altering the scale of random jumps to assess the precision, performance and reliability. Results of obtained data can be visualized in form of figures which shows that for test experiment 1 fitness function is revolving between *10*^−*4*^ to *10*^−*14*^ and *10*^−*6*^ to *10*^−*14*^ for CHSANN and LSANN respectively, Elapsed time in second is found to be within three seconds for both the methods while number of iterations were between *600–1200* and *400–1000* for CHSANN and LSANN respectively. Results of 100 independent runs for test experiments *2–4* can be visualized in Figs 7 and 8, Figs 11 and 12 and Figs 16 and 17 respectively, which demonstrate a similar trend except for the case of nonlinear models for which the maximum elapsed time is found to be 20 seconds due to computational complexity.

#### Comparison with other methods

We compared the proposed techniques in terms of accuracy, elapsed time, ease of calculation and error prediction with the methods presented in [43], [44] and [45]. Methods in [43–45] have been implemented on similar type of problems as in current study. Test example number 3 by Radial basis method presented in [43] and test example no 4 presented in [45] is similar to test experiment no 2 presented in following paper, Test example number 5 presented in [44] is similar to test experiment number 4 presented above and test experiment number 2 presented in [45] is similar to test experiment no 1 by proposed methods.

Following key points of comparison can be noticed.

➢ Radial basis method, method in [44] and method in [45] are providing results on collocation points while proposed schemes are providing a continuous solution.
➢ On the other hand method in [43] is taking 10 to 85 seconds for solving a linear problem while proposed techniques are consuming 6 to 12 seconds and 3 to 5 seconds (Figs 8 and 9) for solving nonlinear problem by ChSANN and LSANN respectively.
➢ Computational complexity of method presented in [43–45] is very large due to solving a large system of nonlinear equations while proposed techniques are too simple in terms of implementation that can be observed through the computational time difference.
➢ There is no way to predict the accuracy in method proposed in [43–45] when there is no exact solution present while proposed schemes can predict accuracy of solution through fitness function. In terms of accuracy methods in [43–45] is giving more accurate results than proposed schemes but limitations of [43–45] is making proposed schemes more powerful.

## Conclusion

In above study we have developed two methods ChSANN and LSANN for simulation of fractional delay differential equation. After analyzing procedure and numerical experiments following points can be concluded.

➢ Proposed methods can be successfully implemented on linear and nonlinear FDDEs with ease of calculation.
➢ Accuracy of method can be increased by improving the learning methodology of NAC.
➢ Accuracy of both the methods is inversely proportional to MSE.
➢ Both the methods can easily handle the nonlinear terms.
➢ Accuracy prediction can be obtained for fractional values of derivatives by observing the MSE values.

In future the proposed schemes can be further developed for accuracy by refining the learning methodology of NAC and by improving the neural architecture. However, it can also be successfully implemented on partial differential equations with some alterations in methodology.
